# Acute hepatitis A-associated aplastic anemia in a pediatric: a case report from Syria

**DOI:** 10.1097/MS9.0000000000003107

**Published:** 2025-03-27

**Authors:** Ammar Albostani, Nour Alhuda Haj Ahmad, Noura Aljnidy, Hibatullah Batal, Othman Hamdan

**Affiliations:** a Faculty of Medicine, University of Aleppo, Aleppo, Syria; b University Pediatrics’ Hospital, Damascus, Syria

**Keywords:** aplastic anemia, bone marrow biopsy, hepatitis A, hepatitis-associated aplastic anemia, immunosuppressive therapy, pediatric

## Abstract

**Introduction and importance:**

Hepatitis A virus is typically an acute infection that is often asymptomatic, especially in children. In Syria, hepatitis A virus infections are not uncommon. However, the emergence of extrahepatic manifestations, such as aplastic anemia, following hepatitis A infection is unpredictable and challenging to manage, with the connection between these conditions remaining poorly understood. This paper aims to highlight this rare manifestation of aplastic anemia linked to hepatitis A infection in children.

**Case presentation:**

We report a rare case of hepatitis-associated aplastic anemia in a 3-year-old Syrian boy who exhibited symptoms of fever, bruising, and mucosal bleeding 1 month after the onset of acute hepatitis. The diagnosis was confirmed via bone marrow examination, and he was treated with immunosuppressives, resulting in full recovery after a year of follow-up with no need for bone marrow transplantation.

**Clinical discussion:**

Hepatitis A infection associated with aplastic anemia is an extremely rare condition. Its etiology might be related to the immune system, and its diagnosis is always confirmed with bone marrow biopsy. Treatment includes hematopoietic cell transplantation if the immunosuppressive therapy is not effective. Further research is essential to understand the mechanisms and optimize treatment options.

**Conclusion:**

Awareness of the potential association between hepatitis A and aplastic anemia is essential for early diagnosis and effective management. More studies are required to enhance our understanding of the condition and improve therapeutic approaches.

## Introduction

Hepatitis A (HA) is an acute viral infection that primarily affects the liver, yet its immune-mediated mechanisms are still not fully understood^[1]^. Although HA can infect individuals of all ages, it typically presents as an asymptomatic or subclinical infection in children. While the disease is usually self-limited, individuals with preexisting liver conditions or older adults may experience more severe symptoms^[2]^.

**HIGHLIGHTS**

- The occurrence of aplastic anemia following hepatitis A infection is uncommon, especially in pediatric cases.
- This study highlights the rare case of a 3-year-old child who developed aplastic anemia following hepatitis A infection.
- Studies on hepatitis-associated aplastic anemia patients reveal immunological abnormalities and positive response to immunosuppressive therapy.
- This case underscores the need for vigilance in monitoring children with Hepatitis A for potential extrahepatic complications, including hematologic abnormalities.

Although rarely fatal, HA can lead to extrahepatic manifestations, and in some cases, serious complications may arise^[2]^. One such rare complication is aplastic anemia (AA), a bone marrow disorder characterized by the failure to produce sufficient red blood cells, white blood cells, and platelets. While AA can occur in individuals of any age or background, it is particularly rare in children and poses significant diagnostic and therapeutic challenges^[3]^.

Hepatitis-associated AA (HAAA) is defined as acute hepatitis followed by pancytopenia occurring a few weeks to months after the onset of hepatitis^[4]^. The occurrence of AA following HA infection is exceedingly rare, particularly in children^[5]^. In this paper, we present a rare case of a 3-year-old child who developed AA 1 month following an HA infection. The report highlights the clinical manifestations, underlying etiology, diagnostic approach, and treatment options for this condition. Additionally, we describe a successful treatment strategy employed in this case, offering valuable insights into the management of such rare and complex presentations. We highlighted the challenges encountered in managing this unique case as per the CARE 2013 Guidelines^[6]^.

## Case presentation

A 3-year-old boy presented to the pediatric clinic with fever, epistaxis, gum bleeding, and recurrent bruising over the past 4 months. His parents reported a previous infection with HA 5 months prior to the visit. Since the onset of these symptoms, the child had been on corticosteroids (2 mg/kg), which were later reduced (1 mg/kg) due to the development of edema. Laboratory results showed a deterioration in blood components (Table 1), necessitating weekly platelet transfusions and biweekly whole blood transfusions. Immunological studies confirmed prior HAV infection through the presence of HAV IgG antibodies.

**Table 1:** Laboratory results of the patient starting at the admission and throughout 1 year of follow-up.

Test	Results at presentation	Results after 1 month	Results after 6 months	Results after 1 year	Ref. range
Hemoglobin	6.9 g/dL	11.4 g/dL	6.4 g/dL	13.6 g/dL	13.5–17.5 g/dL
Hematocrit	20.2%	34.2%	18.6%	38.7%	40–52%
Red blood cell count	2.5 million/mm^3^	3.8 million/mm^3^	2.22 million/mm^3^	4.4 million/mm^3^	4.5–6.5 million/mm^3^
Leucocytes	1360 µL	2870 µL	5400 µL	10 520 µL	4000–10 000 µL
Neutrophils	17.3%	3.5%	22.8%	39.2%	50%–70%
Lymphocytes	70.0%	85.2%	73.1%	55.7%	20%–45%
Monocytes	8.7%	7.4%	3.9%	3.7%	2%–10%
Eosinophils	0.2%	0.1%	0.2%	1.2%	1%–6%
Basophils	3.8%	3.8%	0.0%	0.0%	0%–1%
Platelets	7000 µL	15 000 µL	18 000 µL	240 000 µL	150–450 × 1000/µL
MCV	80.7 fL	89.5 fL	83.8 fL	88.4 fL	80–95 fL
MCH	27.7 pg	29.7 pg	28.8 pg	31.1 pg	27–34 pg
MCHC	34.4 g/dL	33.2 g/dL	34.4 g/dL	35 g/dL	30–35 g/dL
RDW	47.0 fL	38.6 fL	38.2 fL	35.5 fL	35–46 fL
SGPT (ALT)	117 U/L	–	–	–	Up to 45 U/L
Complement C3	129 mg/dL	–	–	–	89–170 mg/dL
Complement C4	23 mg/dL	–	–	–	14–36 mg/dL
IgG	1595 mg/dL	–	–	–	420–1200 mg/dL
IgM	97 mg/dL	–	–	–	45–200 mg/dL
IgA	119.7 mg/dL	–	–	–	18–160 mg/dL
Direct Coombs test	Negative	–	–	–	Negative
Indirect Coombs test	Negative	–	–	–	Negative
Anti-CMV IgG	307 U/mL	–	–	–	Up to 4 U/mL
Epstein–Barr virus (VCA) IgG	58.5 (reactive) index	–	–	–	Index
Anti-HAV IgM (MEIA)	0.3 IU/L (negative)	–	–	–	Up to 1.0 IU/L
Anti-HAV IgG	0.20 IU/L (positive)	–	–	–	Negative: more than 1.0 IU/L

CMV, cytomegalovirus; HAV, hepatitis A virus; MCV, mean corpuscular volume; MCH, mean corpuscular hemoglobin; MCHC, mean corpuscular hemoglobin concentration; MEIA, microparticle enzyme immunoassay; RDW, red cell distribution width; SGPT (ALT), serum glutamic-pyruvic transaminase (Alanine Aminotransferase); VCA, viral capsid antigen.

**Figure 1.**
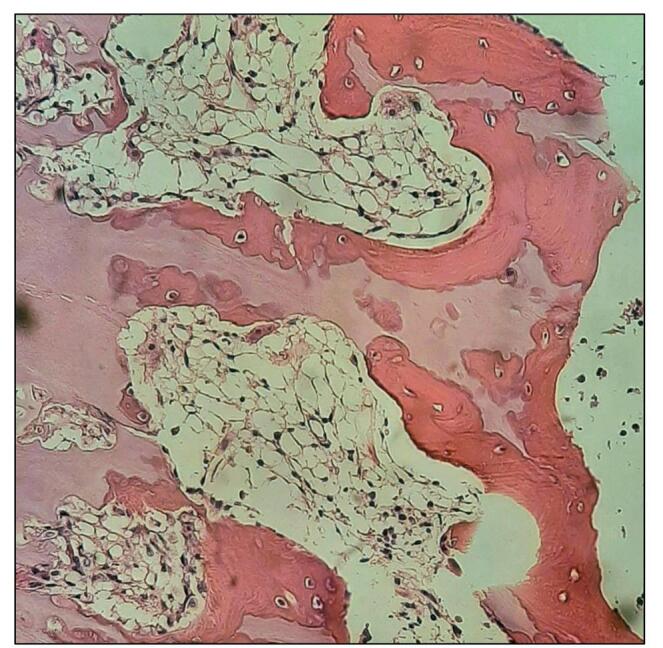
Microscopic image of the patient’s bone marrow, revealing cortical bone and hypocellular marrow spaces which is consistent with aplastic anemia. No neoplastic changes are seen.

Following the laboratory tests, a bone marrow biopsy was obtained for pathological analysis, which revealed markedly hypocellular marrow spaces, consistent with hypoplasia/AA, with no evidence of neoplastic changes (Fig. 1). Based on these findings, the patient was diagnosed with AA secondary to HA. Treatment included a month of eltrombopag (25 mg), followed by cyclosporine, an immunosuppressive agent, with a planned bone marrow transplant if no improvement is seen within a year of follow-up.

**Figure 2.**
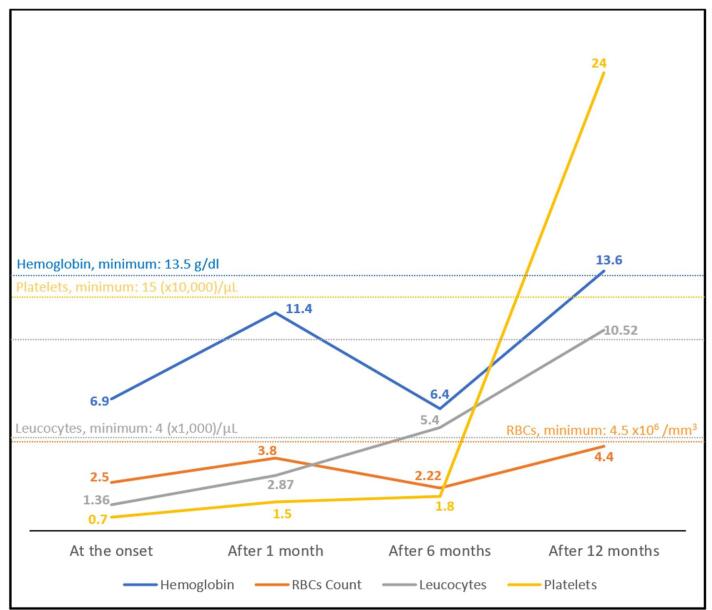
Trends of the main CBC laboratory values during the follow-up. Regard that the platelets count is divided by 10 000, and the leucocyte is divided by 1000 within the chart. CBC, complete blood count; RBCs: red blood cells.

After 1 year of follow-up, our patient achieved full recovery, primarily with cyclosporine (75 mg/day). Symptoms such as bruising, gum bleeding, and fevers have completely disappeared. His laboratory results after 1 year are shown in Table 1 and are almost within or near normal ranges. The trend of each complete blood count (CBC) test result is shown in (Fig. 2).

## Discussion

AA is an uncommon complication associated with hepatitis, most frequently observed following idiopathic hepatitis. It has been reported in conjunction with various hepatitis types, including A, B, and C, as well as other viral infections such as cytomegalovirus, Epstein-Barr Virus (EBV), and parvovirus B19. Notably, HA virus (HAV) accounts for less than 3% of these cases^[4,7]^. Kumar *et al*^[2]^ reviewed the literature and highlighted that hematological complications can occur following HAV infection. Their findings revealed that anemia was the most common complication, present in 25.6% of cases involving hematological abnormalities. Other reported complications included leucopenia in 11.5% of cases, leukocytosis in 12.8%, and thrombocytopenia in 5.1%. Notably, only 8.9% of pediatric patients experienced more than two hematological complications simultaneously. These findings underscore the variability in hematological manifestations associated with HAV infection, particularly in pediatric populations.

The exact mechanism behind HAAA remains uncertain. A study by Gonzalez-Casas *et al*^[8]^ has found various immunological abnormalities in patients with HAAA and noted a positive response to immunosuppressive therapy (IST). These findings suggest that the underlying pathological mechanism in HAAA could be related to the immune system. Fu *et al*^[4]^ demonstrated in their study that the immune response in patients with HAAA is marked by a notable dominance of CD8+ T lymphocytes. This suggests that, in the early stages of hepatitis, cytotoxic T lymphocytes may recognize similar target antigens in both hepatocytes and bone marrow cells. Research studies indicate that patients with AA exhibit a reduced CD4/CD8 ratio in their peripheral blood^[4]^. IST has been shown to restore a normal Gaussian distribution of the T-cell repertoire, likely due to the loss of T cells and antigen clearance. Similarly, HAAA is associated with a significantly low CD4/CD8 ratio and an increased proportion of Human Leukocyte Antigen-DR (HLA-DR+) CD8 cells. These findings suggest that marrow failure in HAAA is driven by CD8 T-cell-mediated destruction^[7]^.

HAAA is twice as prevalent in the Far East compared to the West and is more frequent in populations with lower socioeconomic status^[9]^. In the West, HAAA is reported in 2%–5% of all AA cases. In the Far East, on the other hand, documented cases of HAAA among AA are approximately 4%–10%^[10]^. The Syrian population experiences higher rates of HAV due to limited access to clean water and sanitation, which facilitates the transmission of the infection^[11]^. In fact, 71.2% of all acute hepatitis cases in Syria are attributed to HAV, with the World Health Organization reporting nearly 49 300 HAV cases in 2015^[7]^. Fu *et al*^[4]^ performed a retrospective study in China involving 81 patients with HAAA, comprising 56 males and 25 females, which raises the suspicion that this condition is seen more in males than in females. Alsakkal *et al*^[7]^ reported three case series from Syria with similar characteristics, all involving male patients – two aged 11 years and one aged 17 years. Our patient, being a male pediatric, is comparable to other similar cases reported in the literature as the paper of Botero *et al*^[12]^ presenting a male pediatric patient aged 10 years, with an AA condition after HAV infection.

HAV infection is usually self-limited and asymptomatic, especially in most children under 6 years old. However, the majority of older patients exhibit symptoms such as fatigue (52%–91%), nausea/vomiting (26%–87%), fever (18%–75%), abdominal discomfort (37%–65%), followed by dark urine (28%–94%), jaundice, and, in rare cases, fulminant liver failure, occurring in about 1% of cases and lasting 2–8 weeks with a mortality rate of 0.2%^[1,2]^. According to Altay *et al*^[9]^, when AA is associated with HA, it typically appears within a mean period of 5.06 ± 4.19 months. HAAA presents with variable symptoms, including fever, fatigue, pallor, bacterial infections, and hemorrhagic bruising or petechiae, particularly when signs of bone marrow failure are evident, such as pancytopenia and absolute reticulocytopenia on a CBC^[8]^. As mentioned earlier, our patient displayed typical HAAA manifestations, including high fever, mucosal bleeding, and pancytopenia, confirmed by laboratory results. Notably, the patient developed AA just 1 month after the onset of HAV infection, which is considered relatively rapid. In the case of Botero *et al*^[12]^, the patient displayed AA only 15 days following the initiation of HAV infection. Alsakkal *et al*^[7]^ reported three patients in his case series from Syria, all presented with symptoms similar to our patient. They included fever, fatigue, jaundice color, petechiae, and purpuras.

A microscopic analysis of a bone marrow biopsy typically provides sufficient information to confirm the diagnosis, after ruling out congenital bone marrow failure disorders, malignancies, myelodysplastic syndromes, connective tissue disorders, medications, radiation damage, and other similar factors^[4,8]^. Alsakkal *et al*^[7]^ confirmed all their three-case series with bone marrow biopsies after requesting hematological and serological laboratory tests. The management of HAAA relies on two main options: hematopoietic cell transplantation and IST. The latter serves as the primary alternative for patients without an HLA-matched sibling donor^[4,8]^. In our patient, we suspected pancytopenia due to lab results shown in Table 1 and could only confirm the diagnosis with a bone marrow examination. The patient received whole blood transfusion at the beginning of his clinical manifestations due to the decrease in the formation of all blood contents. Eventually, he received an IST (cyclosporine, 75 mg). We planned a bone marrow transplantation if this approach does not fully resolve his condition. Fortunately, our patient showed major improvement in his laboratory results after 1 year of follow-up, and his previous symptoms, such as fatigue and jaundice, are completely relieved. In HAAA conditions, ISTs have proven their effectiveness in about 70% of patients. However, the other 30% of patients who do not respond to ISTs are recommended to undergo hematopoietic stem cell salvage treatment (i.e., bone marrow transplantation)^[4]^. Our study has a few limitations. First, the availability of resources for funding the patient’s treatment was limited, which may have influenced the management approach. Second, as a case report, our findings are based on a single instance, which limits the generalizability and statistical power of our experience with this condition.

## Conclusion

In conclusion, this study helps in raising clinical awareness about the extrahepatic manifestations of HAV infection and the importance of ongoing monitoring for hematological abnormalities in children with hepatitis to ensure timely and prioritized treatment. HAAA is a rare complication, which presents symptoms such as fatigue, fever, jaundice, and liver failure, with diagnosis confirmed through blood tests and bone marrow biopsy. Routine hematologic monitoring in pediatric HA cases is crucial to avoid delayed diagnosis of complications. ISTs have proven effective due to the immune-mediated etiology, with bone marrow transplantation recommended as a second-line treatment if no improvement is observed. Further research is encouraged to deepen our understanding of this complication and the connection between HAV and extrahepatic hematological manifestations.

## Data Availability

Since all data are included in the manuscript, there is no need for data sharing.

## Research registration unique identifying number (UIN)

Our research study does not involve human subjects.
